# Design and development of a peptide-based adiponectin receptor agonist for cancer treatment

**DOI:** 10.1186/1472-6750-11-90

**Published:** 2011-10-05

**Authors:** Laszlo Otvos, Eva Haspinger, Francesca La Russa, Federica Maspero, Patrizia Graziano, Ilona Kovalszky, Sandor Lovas, Kaushik Nama, Ralf Hoffmann, Daniel Knappe, Marco Cassone, John Wade, Eva Surmacz

**Affiliations:** 1 Temple University, Department of Biology, Philadelphia, PA 19122, USA; 2 Temple University, Sbarro Institute for Cancer Research and Molecular Medicine, Philadelphia, PA 19122, USA; 3 University of Verona, Department of Medical Oncology, 37189 Verona, Italy; 4 Semmelweis University Medical School, 1st Department of Pathology and Experimental Cancer Research, 1085 Budapest, Hungary; 5 Creighton University, Department of Biomedical Sciences, Omaha, NE 68178, USA; 6 Leipzig University, Institute of Bioanalytical Chemistry, Leipzig 04103, Germany; 7 Florey Neuroscience Institutes, Melbourne, 2010 Victoria, Australia

## Abstract

**Background:**

Adiponectin, a fat tissue-derived adipokine, exhibits beneficial effects against insulin resistance, cardiovascular disease, inflammatory conditions, and cancer. Circulating adiponectin levels are decreased in obese individuals, and this feature correlates with increased risk of developing several metabolic, immunological and neoplastic diseases. Thus, pharmacological replacement of adiponectin might prove clinically beneficial, especially for the obese patient population. At present, adiponectin-based therapeutics are not available, partly due to yet unclear structure/function relationships of the cytokine and difficulties in converting the full size adiponectin protein into a viable drug.

**Results:**

We aimed to generate adiponectin-based short peptide that can mimic adiponectin action and be suitable for preclinical and clinical development as a cancer therapeutic. Using a panel of 66 overlapping 10 amino acid-long peptides covering the entire adiponectin globular domain (residues 105-254), we identified the 149-166 region as the adiponectin active site. Three-dimensional modeling of the active site and functional screening of additional 330 peptide analogs covering this region resulted in the development of a lead peptidomimetic, ADP 355 (H-DAsn-Ile-Pro-Nva-Leu-Tyr-DSer-Phe-Ala-DSer-NH_2_). In several adiponectin receptor-positive cancer cell lines, ADP 355 restricted proliferation in a dose-dependent manner at 100 nM-10 μM concentrations (exceeding the effects of 50 ng/mL globular adiponectin). Furthermore, ADP 355 modulated several key signaling pathways (AMPK, Akt, STAT3, ERK1/2) in an adiponectin-like manner. siRNA knockdown experiments suggested that ADP 355 effects can be transmitted through both adiponectin receptors, with a greater contribution of AdipoR1. *In vivo*, intraperitoneal administration of 1 mg/kg/day ADP 355 for 28 days suppressed the growth of orthotopic human breast cancer xenografts by ~31%. The peptide displayed excellent stability (at least 30 min) in mouse blood or serum and did not induce gross toxic effects at 5-50 mg/kg bolus doses in normal CBA/J mice.

**Conclusions:**

ADP 355 is a first-in-class adiponectin receptor agonist. Its biological activity, superior stability in biological fluids as well as acceptable toxicity profile indicate that the peptidomimetic represents a true lead compound for pharmaceutical development to replace low adiponectin levels in cancer and other malignancies.

## Background

Adiponectin is a relatively large (244 amino acid) cytokine normally produced by the fat tissue and found in human serum at concentrations of 2-20 μg/mL [1-5]. Circulating adiponectin levels are inversely correlated with body mass index (BMI) [6]. Adiponectin is considered a protective hormone exhibiting beneficial effects against insulin resistance, cardiovascular disease, inflammatory conditions, and cancer [5-11].

Adiponectin circulates in trimeric, hexameric, and higher order complexes [12]. The C-terminal half of protein representing the globular domain (gAd) exhibits potent metabolic effects in various tissues [13-15]. Two adiponectin receptors have been identified, AdipoR1 and AdipoR2. Both receptors are 7-channel integral membrane proteins containing an N-terminal intracellular portion and a C-terminal extracellular-transmembrane domain [16,17]. AdipoR1 is a high-affinity receptor for gAd and a low affinity receptor for the full-size ligand [18]. AdipoR1 has 4 very short extracellular domains composed of 13, 6, 11 and 16 residues, respectively [17].

The major intracellular signal induced by adiponectin is the energy-sensing AMP-activated protein kinase (AMPK) pathway [13,19]. However, some adiponectin-dependent effects appear to be AMPK-independent [20]. In addition, adiponectin can modulate in a tissue context-dependent manner several other signaling effectors, such as extracellular-signal-regulated kinases 1 and 2 (ERK1/2), p38 kinase, peroxisome proliferator-activated receptor-α (PPARα), stress-responsive c-Jun N-terminal kinase (JNK), Wnt receptor, nitric oxide (NO), signal transducer and activator of transcription 3 (STAT3) factor, nuclear factor-κB (NF-κB), and ceramide [19-29]. Targeted disruption experiments suggested that AdipoR1 transmits signals mainly through AMPK, while AdipoR2 acts through PPARα-related pathways [19].

Recent evidence implicated adiponectin in the prevention of cancer [4,30,31]. Epidemiological studies found an inverse correlation between adiponectin and the risk of developing several obesity-related malignancies, including cancers of the breast, endometrium, colon, and prostate [7,32-34]. The best-documented associations in breast cancer show that adiponectin levels are reduced in cancer patients vs. controls [34-36], and low adiponectin levels correlate with more aggressive tumors and higher frequency of lymph node metastasis [10,37]. In agreement with this, *in vitro *studies demonstrated that adiponectin or its globular form can inhibit the proliferation of breast, colorectal and prostate cancer cells [1,26,38-44].

Depending on the experimental model, cytostatic/apoptotic effects of adiponectin can be associated with an increased activation of AMPK, reduced ERK1/2 signaling [40], inhibition of the Akt kinase and glycogen synthase kinase/β-catenin pathway [45], and/or enhanced expression of Bax and p53 pro-apoptotic genes [44]. In addition, adiponectin can also reduce cancer cell migration and invasion [46]. In animal models, adiponectin suppresses the growth of T47D and MDA-MB-231 breast cancer xenografts, and in some cases, inhibits tumor neoangiogenesis [45,47].

The adiponectin receptors, AdipoR1 and AdipoR2, have been detected in human breast cancer specimens, but not clearly associated with other biomarkers [26,48-50]. AdipoR1 appears to play a more definite role in breast cancer, as adiponectin-dependent antiproliferative effects are abolished by siRNA knockdown of this receptor [1,51]. However, in colon cancer cells, both AdipoR1 and AdipoR2 can transmit cytostatic effects [52]. While data on AdipoR1/2 expression in other malignancies are limited, the receptors have been found in normal colon and colon cancer tissue [53] as well as in gastrointestinal stromal tumors [54].

Although some anti-diabetic drugs (e.g., metformin, a biguanide) [55-57] as well as caloric restriction [58,59] can partially mimic adiponectin action and induce AMPK signaling in cancer tissues, specific and selective compounds targeting AdipoR still await development. At present, adiponectin-based therapeutics are not available, partly due to difficulties in converting the full size adiponectin protein into a viable drug. Here we report on the design and initial preclinical development of adiponectin-based peptide compounds acting as AdipoR agonists in cancer cells.

## Methods

### Initial model building

The three-dimensional (3D) structure of the globular domain (residues 105-254) of human adiponectin was obtained with the YASARA molecular modeling package (Ver. 10.10.29) [60]. The hm_build.mcr macro of the YASARA package with default parameters, except the maximum oligomerization state set to one, was used to build the model. YASARA identified a protein with protein databank (PDB) [61] i.d. 1C3H, which corresponds to a murine isoform of adiponectin, as a sole template. The model was subjected to further refinement using the md_refine.mcr macro of YASARA and to 1 ns constant temperature (300 K) and pressure (1 bar) molecular dynamics (MD) simulations using the AMBER03 force field. Simulation parameters were kept at the values defined by the macro. The structure of the protein was simulated in an 8 × 8 × 8 nm rectangular box with periodic boundaries and endcapping with an *N-*acetyl protecting group to preserve the electronic structure of the backbone. The box containing the protein was filled with 8390 water molecules, 48 Cl^- ^and 53 Na^+ ^ions. The final structure from the simulation was used as a starting parameter to structure calculations of peptide 25 and its derivative, ADP 355.

### Detailed molecular dynamics simulations of peptides

MD simulations were performed with the GROMACS 4.0.7 software package [62] using the OPLS-AA/L force field [63]. Peptides were solvated with 2457 water molecules and one chloride ion to neutralize the charge of the system. The solvated structures were energy minimized by the steepest descent method. Simulations ran for 500 ps at 300 K. At 500.5 ns the simulations continued at 1 bar pressure by coupling the system to external heat and pressure bath. Snapshots of the trajectories were saved at every 0.1 ns. The first 0.5 ns point was considered as equilibration period and was not used for subsequent analysis. A reaction-field correction was used for long-range electrostatic interactions, and an energy dispersion correction was implemented. Trajectories were submitted to cluster analysis using the GROMOS method [64]. Peptide conformations were examined using the middle structure of the largest cluster and using the DSSP program [65]. The root-mean-square deviation (RMSD) of the backbone of the peptide structures was calculated using the g_rms utility of GROMACS.

### Peptide array

A panel of 66 overlapping peptides, each 10 amino acid-long, covering the entire globular domain of the human adiponectin protein was synthesized on a β-alanine derivatized cleavable cellulose membrane [66]. Each consecutive peptide was shifted by 2 residues along the sequence. Another panel of 330 peptides representing analogs of the 66 original peptides was assembled on the same membrane support. In these 10-residue-long overlapping peptides, residues 1, 4, 7 and 10 of the first array were replaced with non-natural amino acids. The substituting amino acids were norvaline for aliphatic residues, D-asparagine for residues with amide-side chain, D-serine for hydroxy-amino acids, D-lysine for cationic residues and 1-amino cyclopentane carboxylic acid for aromatic residues and proline. All peptide sequences are listed in Additional file 1.

The peptides were assembled by Fmoc-synthesis techniques [67], individually cut from the solid support and cleaved from the cellulose membrane by using 2% aqueous triethyl amine overnight [68]. Peptides 23-27 and their modified analogs were purified by reversed-phase high performance liquid chromatography (RP-HPLC) and characterized by matrix-assisted laser desorption/ionization mass spectroscopy (MALDI-MS).

### Synthesis and purification of individual peptides

The adiponectin-based peptide 25, a six-residue middle fragment of peptide 25, and peptidomimetic ADP 355 as well as biotin-labeled analogs of the AdipoR1 extracellular loops were synthesized on the solid-phase by using a CEM Liberty microwave-assisted peptide synthesizer and utilizing Fmoc-chemistry [67]. Biotin was coupled to the peptide while still attached the solid-phase carrier. After cleavage with 95% aqueous trifluoroacetic acid (TFA) containing 2% thioanisole, the peptides were purified by RP-HPLC. MALDI-MS verified the high purity of the peptide preparations. After purification, ADP 355 was lyophilized twice from 2% aqueous acetic acid solution prior to cellular efficacy studies.

### Screening of AdipoR1/peptide binding

The 66 unmodified adiponectin array peptides were individually dried down to wells of an ELISA plate, and tested for binding to biotin-labeled linear synthetic models of the 4 extracellular loops of AdipoR1. The receptor/peptide interaction was detected by horseradish-peroxidase conjugated streptavidin.

### *In vitro *screening of adiponectin-based peptides

Biological activity of the peptides was first assessed using MCF-7 breast cancer cells that are known to express AdipoR1 [38]. MCF-7 cell line was obtained from ATCC (Manassas, VA) and routinely grown in DMEM:F12 plus 5% calf serum (Cellgro Mediatech, Manassas, VA) at 37°C, 5% CO_2_. For screening experiments, MCF-7 cells were plated in 24-well plates at the concentration of 30,000 cells/well. After 12 h of culture in the growth medium, the cells were synchronized in serum-free medium (SFM) (DMEM:F12 supplemented with 0.42 g/mL bovine serum albumin, 1 mM FeSO_4 _and 2 mM L-glutamine) for 24 h, and then shifted back to the full growth medium containing either gAd (Phoenix Secretomics, Burlingame, CA) at 50 ng/mL, individual peptides, or no test compounds. After 24 h, the cells were counted under the microscope with trypan-blue exclusion. Each experiment was performed in triplicate and repeated at least three times.

The array-extracted peptides (1, 2, 3, 19, 20, 21, 22, 23, 24, 26, 27, 28, 29, 30, 31, 37, 55, 56, 57, 58, 59, 60) solubilized at 65°C for 30 min were tested at an approximate concentration of 8-50 ng/mL. In addition, the following peptides: 22 (and its modifications 88, 154, 220, 286, 352), 23 (and its modifications 89, 155, 221, 287, 353), 24 (and its modifications 90, 156, 222, 288, 353), 25 (and its modifications 91, 157, 223, 289 and 355), 26 (and its modifications 92, 158, 224, 290, 356), 27 (and its modifications 93, 159, 225, 291, 357), 28, 29, 30 were further purified by RP-HPLC and screened together with the individually synthesized adiponectin peptides at the exact concentration of 50 ng/mL.

### Cell growth experiments

The effects of the lead active peptide 355 (ADP 335) on cell proliferation were tested in breast cancer cell lines (MCF-7, MDA-MB-231) and in glioblastoma cells (LN18), all obtained from ATCC and routinely cultured as described previously [69-71]. The peptide was tested at 10 pM-100 μM concentrations under conditions described above under peptide screening.

### Detection of AdipoR1 and AdipoR2 and signaling analysis

AdipoR1 and AdipoR2 were detected by Western immunoblotting (WB) using 100 μg of total proteins isolated from growing cell cultures, as described by us previously [72,73] using goat polyclonal AdipoR1 M18 Ab and goat polyclonal AdipoR2 C12 Ab (Santa Cruz Biotechnology, Santa Cruz, CA).

Signaling analysis was performed using MCF-7 and MDA-MB-231 breast cancer cells and LN18 glioblastoma cells. The cells at 70-80% confluence were shifted to SFM for 24 h, then SFM was removed, the cultures were washed 2 x with PBS, placed in normal growth medium for 1 h, and then treated with ADP 355 at 100 nM (MCF-7 cells) or 10 μM (MDA-MB-231 and LN 18 cells) for 0-60 min, while gAd at 50 ng/mL was applied for 60 min only. Untreated cells were used as negative control. After the treatment, the cells were lysed, as previously described [72,73] and 100 μg of proteins were analyzed by WB for the expression of phosphorylated (p) and total forms of several signaling molecules. The following primary Abs from Cell Signaling (Danvers, MA) were used: 1) pAMPKα (T172) D79.5E rabbit mAb 1: 750; 2) total AMPKα rabbit mAb 1:1000; 3) p44/42 MAPK (T202/Y204) rabbit mAb 1:1000; 4) total 44/42 MAP kinase rabbit mAb 1:1000; 5) pSTAT3 (Y705) D3A7 rabbit mAb 1: 500; 6) total STAT3 79D7 rabbit mAb 1:2000; 7) pAkt (Ser 473) rabbit mAb 1:1000; 8) total Akt rabbit mAb 1:1000. Protein loading was verified by evaluating the expression of a constitutive enzyme glyceraldehyde-3-phosphate dehydrogenase (GAPDH) using 6C51 mAbs 1:1000 (Santa Cruz). The following secondary Abs (Santa Cruz) were used where appropriate: 1) donkey anti-goat IgG-HRP; 2) goat anti-mouse IgG-HRP; 3) goat anti-rabbit IgG-HRP, all applied at 1:1000 dilution. The intensity of specific protein bands was quantified by the ImageJ software (distributed by the National Institutes of Health, Bethesda, USA). The ratio of phosphorylated to total levels was calculated for each protein, and expressed as % change vs. untreated controls (taken as 100%).

### siRNA experiments

MCF-7 cells expressing approximately equal levels of AdipoR1 and AdipoR2 were used to assess the contribution of each receptor in the response to ADP355. AdipoR1 and AdipoR2 siRNA as well scrambled siRNA were purchased from Santa Cruz Biotechnology. Dilution of siRNA reagents and transfection of cells was performed following the manufacturer's protocol. For growth experiments, the cells were plated in 24-well plates at 50 × 10^4 ^cells/well and transfected with 5 μl of 10 μM stock siRNA. For WB, the cells were grown in 60 mm plates at 5 × 105 cells/plate and transfected with 15 μl of 10 μM stock siRNA. The cells were processed for WB or treated with ADP355 at 48 h following transfection.

### Peptide stability in mouse blood and mouse serum

Sixty μg of ADP 355 were dissolved in 100 μL water, and 10 μL aliquots were mixed with 100 μL of freshly drawn mouse blood. After 30 min of incubation at 37°C, blood cells were centrifuged at 10,000 × g. Fifty μL serum was mixed with 50 μL phosphate buffered saline pH 6.8 (PBS), and serum proteins were precipitated by addition of 45 μL aqueous 15% trichloroacetic acid (TCA) for 10 min at 4°C. After centrifugation at 12,000 × g, the supernatant was neutralized with 0.1 M aqueous sodium hydroxide and 0.5 μL of this solution was mixed with 0.5 μL α-cyano-4-hydroxycinnamic acid (4 mg/mL in 60% aqueous acetonitrile containing 0.1% TFA) as matrix on a sample plate. Analysis was performed using a MALDI time-of-flight tandem mass spectrometer (MALDI-TOF/TOF-MS, 4700 proteomic analyzer, Applied Biosystems, Weiterstadt, Germany). Additionally, the neutralized supernatant was loaded on a Jupiter C_18 _RP-HPLC column (4.6 mm internal diameter, 150 mm length, 5 μm particle size, 30 nm pore size) previously calibrated with known amounts of ADP 355 dissolved in PBS. Absorbance was measured at 214 nm.

ADP 355 was also incubated at 37°C with 25% aqueous mouse serum at a final concentration of 150 μg/mL. After 0, 15, 30, 60, 120, and 240 min, 95 μL aliquots were mixed with 25 μL 15% aqueous TCA and were incubated for 10 min at 4°C. Sample analysis followed the protocols described above.

### *In vivo *activity of ADP 355 in an orthotopic xenograft breast cancer model

Ten 8-week-old female immunocompromised (*scid) *mice (genetic background CB17/Icr) were anesthetized to allow the implantation of 2.5 × 10^6 ^MCF-7 cells into the two inguinal mammary glands. When tumors were palpable in all animals (34 days after cell transplantation), the mice were divided into 2 groups containing animals with comparable tumor sizes. One group of 5 mice was treated daily with 1 mg/kg peptide ADP 355 intraperitoneally (ip), while the other group of 5 mice reminded untreated. After 28 days of treatment, the animals were killed by CO_2 _inhalation, and tumors were carefully removed, photographed and weighed. All vertebrate animals of this study were maintained and handled in accordance with the recommendations of the Guidelines for the Care and Use of Laboratory Animals and were approved by the Animal Care Committee of Semmelweis University (permission No.:399/003/2005).

### *In vivo *toxicity

ADP 355 was injected into 4 groups of three 10-12 week old female CBA/J mice. Bolus ip doses were administered at 5 mg/kg, 10 mg/kg, 25 mg/kg or 50 mg/kg in sterile saline and the animals were observed for signs of systemic toxicity (tremor, head tilt, reduced activity and squinting) for 4 days. On day 5, the mice were sacrificed by CO_2 _inhalation. The potential peptide elimination organs: the livers, spleens and kidneys were removed and weighed. All procedures for vertebrate animal experiments were approved by the Animal Health and Food Control Committee of Budapest, protocol number 399/033/2005.

## Results

### Identification of the active site of adiponectin protein

**Figure 1:**
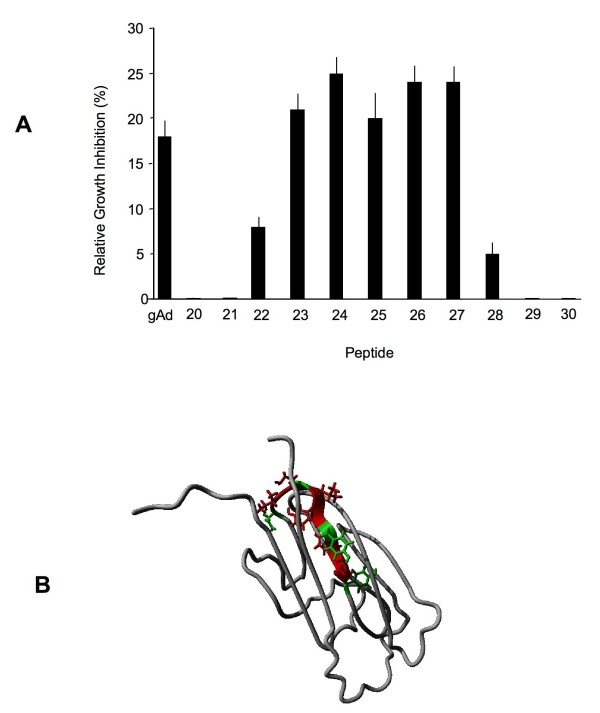
**Identification of the active site of adiponectin**. A) Effects of adiponectin fragments encompassing the active site on the growth of MCF7 cells. The activity of the entire globular domain of adiponectin (gAd) is included for comparison. The data are averages from 3 different assays and represent average results +/- SE and were analyzed by Student t-test, p < 0.05. The sequences of all tested peptides are listed in Additional file 1. B) High-resolution structure of the adiponectin monomer with the peptide 25 and active site amino acid side-chains colored. Conservative substitutions of residues marked in green could be made without loss of biological activity; residues marked with red could not be substituted.

The peptides extracted from the array were tested for cytostatic activity in adiponectin sensitive MCF-7 cells [38]. In control experiments, we used gAd at 50 ng/mL, a concentration that induced maximal growth inhibition in our dose response experiments in MCF-7 cells (data not shown) and has previously been described as cytostatic in breast cancer cells [1]. Peptides 23-27 at 50 ng/mL inhibited MCF-7 cell proliferation by 19-26% relative to untreated controls, while other peptides, either flanking this domain or distant, were ineffective or produced only minimal (3% or less) cytostatic effects (Figure 1A and Additional file 1). gAd restricted MCF-7 cell growth by ~18% (Figure 1A).

The sequence covered by the active peptides 23-27 is: H-Lys-Phe-His-Cys-Asn-Ile-Pro-Gly-Leu-Tyr-Tyr-Phe-Ala-Tyr-His-Ile-Thr-Val-NH_2_, and this fragment corresponds to amino acids 149-166 of human adiponectin protein (Table 1).

**Table 1:** Summary of structure-function analysis of adiponectin fragments

Original Peptide (aa number in adiponectin)	Cytostatic Activity of Original Peptide vs. gAd (% Increase)	Sequence Modifications of Original Peptide	Cytostatic Activity of Modified Peptides vs. gAd (% Increase)
ADP 23 (149-158)	17	Lys-Phe-His-Cys-Asn-Ile-Pro-Gly-Leu-Tyr * * # *	0-61

ADP 24 (151-160)	39	His-Cys-Asn-Ile-Pro-Gly-Leu-Tyr-Tyr-Phe # # # #	0-40

ADP 25 (153-162)	11	Asn-Ile-Pro-Gly-Leu-Tyr-Tyr-Phe-Ala-Tyr * * * *	21-126

ADP 26 (155-164)	33	Pro-Gly-Leu-Tyr-Tyr-Phe-Ala-Tyr-His-Ile # # # #	0-33

ADP 27 (157-155)	33	Leu-Tyr-Tyr-Phe-Ala-Tyr-His-Ile-Thr-Val # # * #	0-66

Proposed minimal active site: Ile-Pro-Gly-Leu-Tyr-Tyr-Phe-Ala

Conservative substitutions allowed (bolded): **X**-Ile-Pro-**Gly**-Leu-Tyr-**Tyr**-Phe-Ala-**X**

The cytostatic activity of original and modified peptides was evaluated in MCF-7 cells, as described in Methods and was calculated relative to the activity of gAd (baseline). Underlined amino acids indicate the residues where conservative modifications were made. Replaceable residues are marked with * and non-replaceable residues are marked with #. X, non-natural amino acid.

According to the currently most accepted model, the globular domain of human adiponectin is a β-barrel-type structure where the β-sheets are connected with ω-loops. The identified active peptides are located on the loop-β-sheet region of the protein (Figure 1B). While approximately half of the sequence, covering peptides 26 and 27, is located inside the trimer bundle, the N-terminal region falls slightly outside the trimer boundaries. The side-chains of the C-terminal 2/3 of the identified active site are facing outside (Figure 1B). The center of the active peptides has homology only with spastin, immunoglobulin and complement proteins according to a BLAST homology search.

### Identification of minimal adiponectin active site and development of its pharmacologically improved analogs

Next, we generated multiple analogs of peptides 23-27 in order to identify the minimal adiponectin active site as well as introduce chemical modifications improving peptide activity and stability. The activities of all analogs at 50 ng/mL were determined relative to the effects of 50 ng/mL gAd (Table 1).

While peptide 25 was fully active in cell growth inhibition assays, its center 6 residue-long fragment 157-162 did not exhibit any biological activity. Therefore, we generated and tested several longer, 10-residue peptides encompassing the 149-166 adiponectin stretch. We attempted to identify residues in this region that could be freely replaced with non-natural amino acid analogs in order to improve pharmacological properties of the lead peptides. Biological assays identified a highly active short site: Ile-Pro-Gly-Leu-Tyr-Tyr-Phe-Ala, and further structure-function analysis indicated that conservative substitutions in the minimal active site can be introduced at Gly 155 and Tyr 158 residues, without compromising biological activity. Additions of non-natural amino acids at N- and C-termini were envisioned to provide stability against exopeptidase cleavage *in vitro *and *in vivo *(Table 1).

### Identification of ADP 355 as an optimal adiponectin receptor agonist

Biological screening of the analogs of the minimal adiponectin active site resulted in the identification of a peptidomimetic ADP 355 (H-DAsn-Ile-Pro-Nva-Leu-Tyr-DSer-Phe-Ala-DSer-NH_2_) as the most promising adiponectin receptor agonist. The compound is based on the precursor peptide 25 and contains the minimal active site with allowed modifications (Table 1).

### ADP 355 inhibits the growth of AdipoR1/AdipoR2-positive cancer cell lines

In a preliminary, qualitative study, we examined the interaction of peptide 25 to biotin-labeled fragments of the 4 AdipoR1 extracellular loops. The peptide bound the first extracellular loop of AdipoR1, but not other loops (data not shown). BLAST analysis identified an 86% homology between the first loops of AdipoR1 and AdipoR2, suggesting that ADP 355 potentially can interact with both receptors.

**Figure 2:**
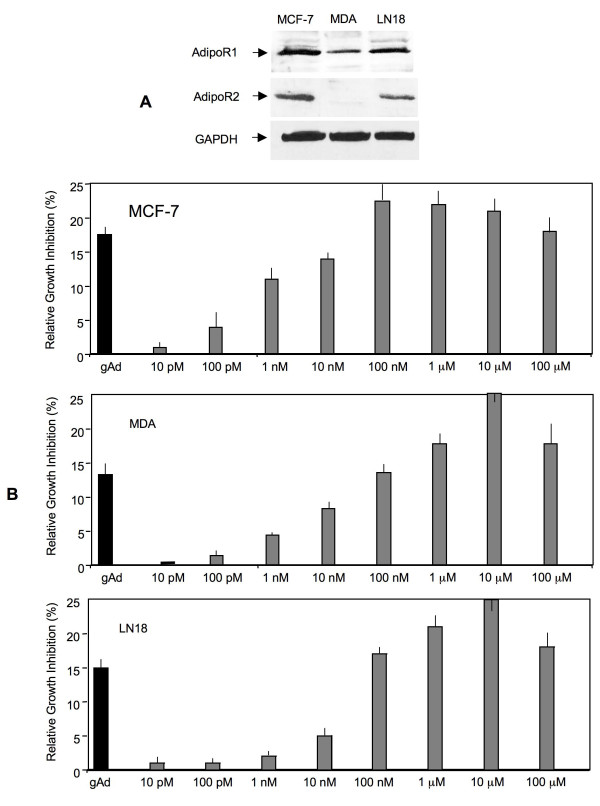
**Effects of ADP 355 on the growth of cancer cells *in vitro***. A) Expression of AdipoR1 (49 kDa) and AdipoR2 (44 kDa) in MCF-7, MDA-MB-231 and LN18 cells was examined by WB, as described in Methods. B) Cytostatic activity of ADP 355 at 10-100 μM was assessed in MCF-7, MDA-MB-231, and LN18 cancer cell lines, as described in Methods. Bars represent % growth inhibition relative to untreated cells +/- SE.

Dose-dependent effects of ADP 355 were tested in different cancer cells lines expressing AdipoR1 and AdipoR2. The highest levels of AdipoR1 were found in MCF-7 cells, while the receptor was less abundant in MDA-MB-231 and LN18 cells (Figure 2A). On the other hand, AdipoR2 was undetectable in MDA-MB-231 cells, and expressed at intermediate and high levels in LN18 and MCF-7 cells, respectively. Preliminary experiments suggested that all cell lines are sensitive to gAd, and the maximal growth inhibition can be achieved with gAd at 50-100 ng/mL (data not shown).

In all cell lines, ADP 355 restricted normal cell growth in a dose-dependent manner. In MCF-7 cells, the best growth inhibition was achieved with ADP 355 at 100 nM-10 μM, while 10 pM-10 nM concentrations were less effective (Figure 2B). In MDA-MB-231 and LN 18 cells, the maximal growth inhibition was noted at 10 μM. In all cell lines, ADP 355 at maximal effective doses produced greater cytostatic effects then gAd at 50 ng/mL (Figure 2B).

### Effects of AdipoR1 or AdipoR2 downregulation on ADP355 activity

**Figure 3:**
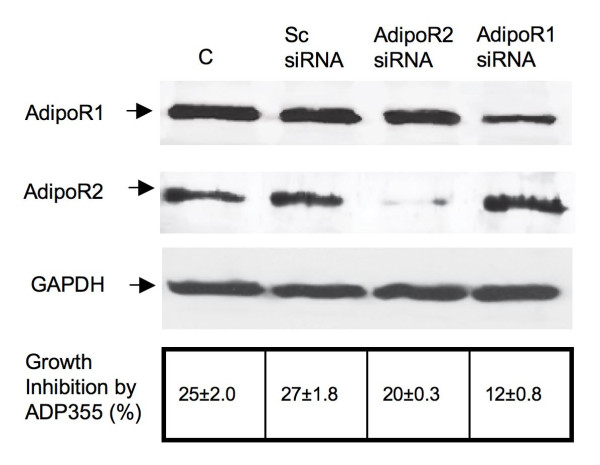
**Effects of siRNA-mediated downregulation of AdipoR1 or AdipoR2 on ADP 355 activity**. The expression of AdipoR1, AdipoR2, and control protein GAPDH were assessed by WB in control (C) cells (transfection medium only), cells treated with scrambled siRNA (Sc siRNA), siRNA targeting AdipoR1, or siRNA targeting AdipoR2, as described in Methods. The relative levels of AdipoR1 and AdipoR2 proteins were calculated by densitometry scanning, as described in Methods, and are provided in Additional file 2. The relative % of growth inhibition upon ADP 355 treatment in cells with different levels of AdipoR1 and AdipoR2 vs. untreated cells is shown in the lower panel table.

To assess the contribution of AdipoR1 and AdipoR2 in mediating ADP 355 effects, we selectively downregulated the expression of each receptor in MCF-7 cells using siRNA technology. The decrease of AdipoR1 by ~60% reduced ADP 355 activity by 52%, while downregulation of AdipoR2 by 90% diminished ADP 355 effects by 20% (Figure 3 and Additional file 2). These results suggested that the peptide can transmit signals through both receptors, but the majority of activity was mediated through AdipoR1. Of note, AdipoR2-negative MDA-MB-231 cells exhibited sensitivity to gAd and ADP355, suggesting that AdipoR1 was sufficient to activate the response.

### ADP 355 differentially modulates AdipoR signaling pathways

**Figure 4:**
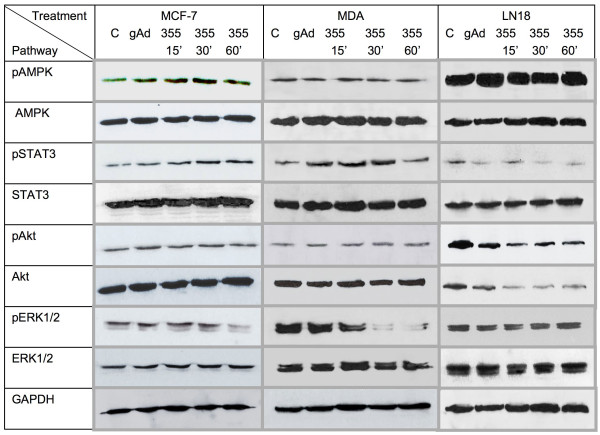
**Effects of ADP 355 on intracellular cell signaling in cancer cells**. The effects of ADP 355 on signaling pathways in MCF-7, MDA-MB-231, and LN18 cells at 0-60 min of treatment were studied by WB, as described in Methods. The expression of GAPDH was used as determination of protein loading. The relative levels of phosphorylated/total proteins were calculated by densitometry scanning, as described in Methods, and are provided in Additional file 3.

We examined the effects of ADP 355 on different adiponectin signaling pathways in MCF-7, MDA-MB-231, and LN18 cells (Figure 4 and Additional file 3). The peptide was used at concentrations that produced maximal cytostatic effects and the treatment was carried out for 0-60 min.

Remarkably, depending on cell line, ADP 355 exerted differential signaling effects. In MCF-7 cells, the peptide increased the phosphorylation of AMPK at 15 and 30 min and decreased ERK1/2 phosphorylation at 30-60 min. ADP 355 did not significantly affect the activation of Akt in these cells, but it increased the phosphorylation of STAT3 at 15-60 min (Figure 4 and Additional file 3). In MDA-MB-231 cells, the major pathway affected by ADP 355 was ERK1/2, which was measurably inhibited at 15-60 min of treatment. The peptide transiently increased STAT3 phosphorylation at 15 and 30 min. In MDA-MB-231 cells, ADP 355 did not stimulate AMPK activation, while Akt phosphorylation was moderately activated at 30-60 min. In LN18 cells, ADP 355 decreased STAT3 phosphorylation at 15-60 min and dramatically downregulated total levels of Akt at 15-60 min. However, the peptide did not significantly affect AMPK in these cells. In all cell lines, gAd regulated signaling pathways similar to ADP 355, however its effects were usually less pronounced (Figure 4 and Additional file 3).

### ADP 355 exhibits superior stability in mouse serum and blood

**Figure 5:**
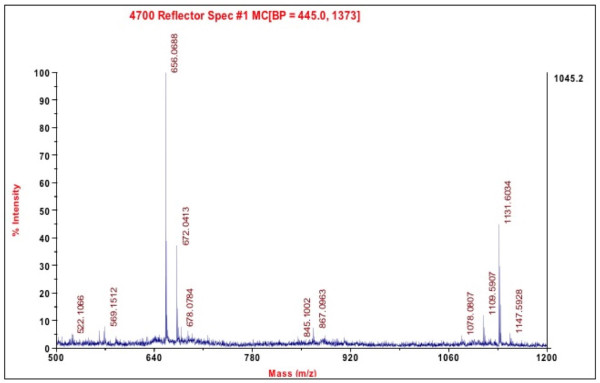
**Stability of ADP 355 in whole mouse blood**. The peptide stability was assessed in whole mouse blood after 30 min of incubation by mass spectroscopy as described in Methods. The only peptide-originated peaks are at 1109 and 1131 M/z, representing the unmodified peptide and its sodium adduct.

Calculating from the degradation rate measured in 25% aqueous serum [74], ADP 355 had a 75 min half-life in mouse serum (data not shown). In whole mouse blood, the peptide was present even after 30 min without noticeable degradation (Figure 5).

### ADP 355 inhibits the growth of MCF-7 xenografts in immunocompromised mice

**Figure 6:**
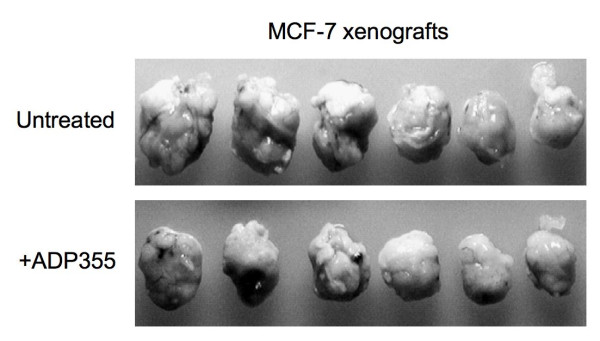
**Anti-tumor ADP 355 activity *in vivo***. Orthotopic MCF-7 xenografts were established as described in Methods. After 34 days, 5 mice were treated with ADP 355 at 1 mg/kg/day dose, and 5 mice remained untreated. After 28 days, the mice were sacrificed and the lesions removed. Due to variability in tumor sizes, the largest and smallest lesions from each group were excluded from the evaluation. The excised, middle-sized lesions, from 3 treated and 3 untreated mice are shown.

The efficacy of ADP355 was assessed in MCF-7 orthotopic xenograft model. The peptidomimetic was injected ip into *scid *mice carrying palpable MCF-7 xenografts at a 1 mg/kg/day dose. After 28 days of treatment, the mice were sacrificed and tumors removed. Due to variability in tumor sizes, the largest and smallest lesions from each group were excluded from the evaluation. Figure 6 shows the remaining 6 tumor lesions (from 3 animals in each group). The total tumor weight in untreated animals was 10.27 g (average tumor weights per mice: 2.56 g, 1,51 g and 1.07 g), and in ADP 355-treated animals 7.10 g (average tumor weights per mice: 1.38 g, 1.36 g and 0.81 g). Thus, ADP 355 therapy reduced established tumor growth by 31% (statistically significant, p < 0.05), relative to untreated controls.

### Preliminary assessment of ADP 355 toxicity *in vivo*

Healthy mice receiving up to 50 mg/kg peptide ADP 355 ip showed no signs of systemic toxicity. Four days after peptide administration the potential peptide elimination organs were removed and weighed. While the spleens of treated and untreated animals were identical in size, the kidney and liver weights were slightly increased in treated mice relative to total body weight at the highest dose of 50 mg/kg (Table 2). Since peptide drugs undergo renal and hepatic clearance [69], an increase of the elimination organ size might indicate the active metabolic processes. In addition, AdipoR1/2, as physiological targets of adiponectin, are found in the liver, and might respond to agonist treatment [75]. However, these limited toxic effects were not observed below the 10 mg/kg dose, a magnitude higher than the therapy dose, identifying ADP 355 as a safe treatment option.

**Table 2:** Toxicity analysis of ADP 355

Peptide dose (bolus ip)	Liver weight (g); relative to total weight (%)	Spleen weight (g)	Kidney weight (g); relative to total weight (%)
Untreated	0.98; 0.054	0.07	0.29; 0.015

5 mg/kg	1.02; 0.054	0.07	0.30; 0.016

10 mg/kg	1.09; 0.059	0.06	0.29; 0.016

25 mg/kg	1.24; 0.059	0.07	0.35; 0.017

50 mg/kg	1.07; 0.062	0.07	0.31; 0.017

CBA/J mice were treated with ADP 355 and toxicity parameters were assessed as described in Methods.

## Discussion

Numerous epidemiological and experimental studies provided evidence linking obesity to an increased risk of developing different malignancies, including breast, colorectal, prostate and endometrial cancers [76-79]. In addition, calorie-rich diet has been shown to induce inflammatory responses in microglia cells, which potentially can promote development of brain neoplasms [80,81].

In obese individuals, especially in those with high visceral fat content, adiponectin levels are low [11]. According to epidemiological studies, low adiponectin levels are associated with elevated cancer risk and development of more aggressive neoplasms [4,11,48]. How exactly adiponectin might prevent or restrict cancer is yet not clear. The relevant mechanisms could involve activation of intracellular metabolic changes similar to those produced by calorie restriction, i.e., stimulation of intracellular signals, such as AMPK, and inhibition of abnormal growth and survival pathways [11]. Thus, pharmacological activation of adiponectin signaling in obese individuals that are refractory to lifestyle modifications could help to restore beneficial pathways normally controlled by this adipokine.

However, development of the whole adiponectin protein as a drug is difficult because of the extreme insolubility of the C-terminal globular domain and its larger peptide fragments. In addition, until now, the adiponectin active site has not been mapped. Consequently, we attempted to generate small peptides that would produce biological effects similar or superior to that of gAd, but would be suitable for pharmaceutical modifications.

First, using peptide arrays and biological screening assays, we mapped the adiponectin active site to amino acids 149-166 within the globular domain of the whole adipokine (Figure 1A). In parallel experiments, we found that peptides covering the active site displayed high affinity to an extended version of the AdipoR1 loop 1 (sequence: Arg-Pro-Asn-Met-Tyr-Fen-Met-Ale-Pro-Leu-Gln-Glu-Lys-Val-Val) that shares 86% homology with the loop 1 in AdipoR2. Further modifications of the active site, followed by structure-function screening resulted in the development of the lead peptidomimetic, ADP 355, as optimal AdipoR agonist.

**Figure 7:**
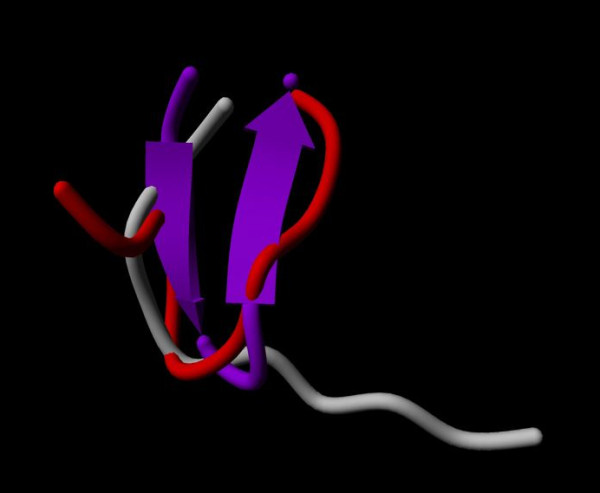
**ADP 355 energy analysis**. Representative energy minimized structures of peptide 25 (red) and ADP 355 (purple) overlaid to the conformation of the 153-162 sequence found in adiponectin protein (grey).

The identified active site of adiponectin can be characterized as a turn region followed by a β-pleated sheet fragment (Figure 1B). When removed from the protein environment, MD studies indicated that the isolated native peptide 25 loses the β-pleated sheet character and forms a series of turns (Figure 7). During MD simulations, the initial turn- β-sheet structures of both peptide 25 and ADP 355 peptides were substantially changed and showed high flexibility. The backbone RMSD values fluctuated with high frequency between 0.1 and 0.7 mm. However, in the case of ADP 355, from 80 ns to 250 ns, the RMSD remained around 0.6 nm, indicting that the peptidomimetic folded into a more stable conformation characterized by a hairpin incorporating almost the entire peptide. In the cluster analysis, the most populated cluster of the peptidomimetic contained more than twice as many structures as the native fragment (31.6% vs 12.4%). If the dominant β-hairpin structure is indeed the active conformation, the significantly increased population of this conformer can explain the improved *in vitro *activity of ADP 355 relative to that of its precursor peptide 25.

Functional assays with ADP 355 demonstrated that the peptide restricts cancer cell proliferation in a dose-dependent manner at 100 nM-10 μM concentrations. In all studied cell lines, this growth inhibition was superior to that obtained with gAd (Figure 2B). Cytostatic activity of ADP 355 is in agreement with several other reports showing similar effects of adiponectin or gAd in cancer models [26,44,45,51,55,82,83]. However, some studies failed to demonstrate any anti-neoplastic activity of this adipokine [54]. These discrepancies likely reflect differences in experimental design as well as cell context, including differential levels of AdipoR1/2 and signaling proteins. Indeed, our work clearly suggests that the levels of AdipoR1 and AdipoR2 vary among cell lines. Some previous reports suggested that cytostatic effects of adiponectin in breast cancer cells are primarily mediated through AdipoR1 [51], and our results with AdipoR2-negative cells and AdipoR2-knockdown cells confirm this notion.

Our signaling studies further confirmed that cell response to adiponectin or its derivatives may be cell-specific. We demonstrated that cytostatic effects ADP 355 coincided with the modulation of specific adiponectin signals that have been associated with growth or survival control, i.e., AMPK, Akt, ERK1/2, and STAT3. Interestingly, the major metabolic adiponectin pathway--AMPK was transiently induced only in MCF-7 cells, while in MDA-MB-231 and LN18 cells, the peptide or gAd did not have any effects (Figure 4).

In MCF-7 cells, ADP 355, but not gAd, decreased ERK1/2 signaling. STAT3 was activated in this cell line by both ADP 355 and gAd. In MDA-MB-231 cells, like in MCF-7 cells, ADP 355 decreased ERK1/2 activation and transiently increased STAT3 signaling. In both breast cancer cell lines, ADP 355 did not affect the major growth/survival Akt pathway. In contrast, ADP 355 and gAd significantly inhibited Akt and STAT3 signals in LN18 cells. Interestingly, the effects on Akt concerned total levels of the enzyme, suggesting that ADP 355 might affect its turnover.

Published data on adiponectin signaling in cancer cells seem to support the notion that the cytokine might induce different signaling pathways in different cell lines. For instance, in many cancer cell lines (breast MCF-7, MDA-MB-231, T47D; colorectal HT-29, CaCO2, SW480; prostate PC3) adiponectin activated AMPK [26,40,54,55]. On the other hand, adiponectin either reduced or did not affect ERK1/2 in MCF-7 or MDA-MB-231 cells, but stimulated the pathway in some colorectal cancer cell lines [1,40,54]. Akt was inhibited by adiponectin in MDA-MB-231 breast cancer cells, but activated in prostate cancer cells LNCaP [82,84]. The upregulation of AMPK and reduction of Akt in response to adiponectin in MDA-MB-231 cells [82] is in contrast with our study and might be related to significantly lower gAd and ADP 355 concentrations used in our experiments, while high doses used by Kim et al. were toxic in our system. Consistent with our results, moderate STAT3 stimulation by adiponectin was noted in MDA-MB-231 cells, while the transcription factor was inhibited in DU145 prostate cancer cells [1,25]. These differences, in part, could reflect variable experimental settings, such as baseline growth conditions, adiponectin reagents used as well as treatment timing and dosage.

To further assess the efficacy of ADP 355, we carried out a preliminary *in vivo *study. In *scid *mice carrying MCF-7 orthotopic xenografts, ADP 355 treatment reduced the growth of established tumors by ~31%, validating AdipoR as a target for breast cancer therapy.

## Conclusions

Here we report on the design and development of a first-in-class AdipoR agonist. AdipoR agonists are viewed as future drugs to treat multiple diseases related to obesity and insulin resistance. The biological activity of our novel ADP 355, including its *in vivo *efficacy, its superior stability in biological fluids, as well as acceptable toxicity profile and low production costs indicate that the peptidomimetic represents a true lead compound for ensuing pharmaceutical development.

## Authors' contributions

LO conceived the study, participated in its coordination and manuscript writing; EH, FLR, and FM analyzed biological activity and intracellular signaling of peptides and peptidomimetics; PG performed siRNA experiments; KN carried peptide cleavage, purification and measurements; DK and RH analyzed peptide stability in biological fluids; IK carried out and analyzed all animal experiments; SL performed all energy calculations; JW synthesized the peptides in solution; ES conceived the study, participated in its design and coordination, and drafted the manuscript. All authors read and approved the final manuscript.

## Supplementary Material

Additional file 1 **Sequences of tested adiponectin-derived peptides**. Designation and amino acid sequences of tested adiponectin-derived peptides. Click here for file

Additional file 2 **Quantification of AdipoR1 and AdipoR2 amounts**. Densitometry quantification of AdipoR1 and AdipoR2 levels following targeted siRNA knockdown experiments. Click here for file

Additional file 3 **Quantification of signaling pathways in ADP 355-treated cancer cells**. Densitometry quantification of pAMPK/AMPK, pSTAT3/STAT3, pAkt/Akt, pERK 1/2/ERK1/2 levels in MCF-7, MDA-MB-231, and LN18 cells treated with gAd or ADP 355. Click here for file
