# Supplementary material for: Design and development of a peptide-based adiponectin receptor agonist for cancer treatment
Source: BMC Biotechnol. 2011 Oct 5;11:90. doi: 10.1186/1472-6750-11-90 (PMC3198688; doi:10.1186/1472-6750-11-90)
Supplement: Additional file 1 — Sequences of tested adiponectin-derived peptides. Designation and amino acid sequences of tested adiponectin-derived peptides. [file 1472-6750-11-90-S1.DOC]

**Additional file 1**

23: (amino acids: 149-158): H-Lys-Phe-His-Cys- Asn-Ile-Pro-Gly-Leu-Tyr-NH_2_

89: same as 23 except D-Lys1 and D-Ser10 (for Lys and Tyr)

155: same as 23 except D-Ser4 (for Cys)

221: same as 23 except Cpc7 (for Pro)

287: same as 23 except D-Ser4 and Cpc7 (for Cys and Pro)

353: same as 23 except D-Lys1, D-Ser4, Cpc7, and D-Ser10 (for Lys, Cys, Pro and Tyr)

Cpc: 1-amino cyclopentane carboxylic acid

24: (amino acids: 151-60): H-His-Cys- Asn-Ile-Pro-Gly-Leu-Tyr-Tyr-Phe-NH_2_

90: same as 24 except D-Lys1 and Cpc10 (for His and Phe)

156: same as 24 except Nva4 (for Ile)

222: same as 24 except Nva7 (for Leu)

288: same as 24 except Nva4 and Nva7 (for Ile and Leu)

354: same as 24 except D-Lys1, Nva4, Nva7, and Cpc10 (for His, Ile, Leu and Phe)

Nva: norvaline

25 (amino acids 153-162): H-Asn-Ile-Pro-Gly-Leu-Tyr-Tyr-Phe-Ala-Tyr-NH_2_

91: same as 25 except D-Asn1 and D-Ser10 (for Asn and Tyr)

157: same as 25 except Nva4 (for Gly)

223: same as 25 except D-Ser7 (for Tyr)

289: same as 25 except Nva4 and D-Ser7 (for Gly and Tyr)

355: same as 25 except D-Asn1, Nva4, D-Ser7, and D-Ser10 (for Asn, Gly, Tyr and Tyr)

26 (amino acids 155-164): H-Pro-Gly-Leu-Tyr-Tyr-Phe-Ala-Tyr-His-Ile-NH_2_

92: same as 26 except Cpc1 and Nva10 (for Pro and Ile)

158: same as 26 except D-Ser4 (for Tyr)

224: same as 26 except Nva7 (for Ala)

290: same as 26 except D-Ser4 and Nva7 (for Tyr and Ala)

356: same as 26 except Cpc1, D-Ser4, Nva7, and Nva10 (for Pro, Tyr, Gly and Ile)

27 (amino acids 157-166): H-Leu-Tyr-Tyr-Phe-Ala-Tyr-His-Ile-Thr-Val-NH_2_

93: same as 27 except Nva1 and Nva10 (for Leu and Val)

159: same as 27 except Cpc4 (for Phe)

225: same as 27 except D-Lys7 (for His)

291: same as 27 except Cpc4 and D-Lys7 (for Phe and His)

357: same as 27 except Nva1, Cpc4, D-Lys7, and Nva10 (for Leu, Phe, His and Val)

All peptides above contained a C-terminal beta-alanine.

Three additional peptides were made and tested that did not contain beta-alanine:

25 (amino acids 153-162): H-Asn-Ile-Pro-Gly-Leu-Tyr-Tyr-Phe-Ala-Tyr-NH_2_

355: same as 25 except D-Asn1, Nva4, D-Ser7, and D-Ser10 (for Asn, Gly, Tyr and Tyr)

Middle 6 of 25: H-Pro-Gly-Leu-Tyr-Tyr-Phe -NH_2_ (this last one was inactive)
