# Supplementary material for: Design and development of a peptide-based adiponectin receptor agonist for cancer treatment
Source: BMC Biotechnol. 2011 Oct 5;11:90. doi: 10.1186/1472-6750-11-90 (PMC3198688; doi:10.1186/1472-6750-11-90)
Supplement: Additional file 2 — Quantification of AdipoR1 and AdipoR2 amounts. Densitometry quantification of AdipoR1 and AdipoR2 levels following targeted siRNA knockdown experiments. [file 1472-6750-11-90-S2.DOC]

Additional file 2

|  | Control | Sc siRNA | AdipoR2  siRNA | AdipoR1  siRNA |
| --- | --- | --- | --- | --- |
| AdipoR1 | 100 | 87 | 78 | 40 |
| AdipoR2 | 100 | 106 | 10 | 143 |
| GAPDH | 100 | 99 | 109 | 102 |

The cells were treated with siRNA, control siRNA, or transfection medium only, as described in Figure 3 legend.The levels of proteins were measured by densitometry scanning of WB, as described in Materials and Methods. The results are average from at least 2 experiments.
