# Supplementary material for: Design and development of a peptide-based adiponectin receptor agonist for cancer treatment
Source: BMC Biotechnol. 2011 Oct 5;11:90. doi: 10.1186/1472-6750-11-90 (PMC3198688; doi:10.1186/1472-6750-11-90)
Supplement: Additional file 3 — Quantification of signaling pathways in ADP 355-treated cancer cells. Densitometry quantification of pAMPK/AMPK, pSTAT3/STAT3, pAkt/Akt, pERK 1/2/ERK1/2 levels in MCF-7, MDA-MB-231, and LN18 cells treated with gAd or ADP 355. [file 1472-6750-11-90-S3.DOC]

**Additional file 3**

MCF-7

| Protein/activation vs. control (%) | gAd | ADP 15’ | ADP 30’ | ADP 60’ |
| --- | --- | --- | --- | --- |
| pAMPK/AMPK | +47 (161/109) | +106 (206/100) | +143 (253/104) | +43 (181/126) |
| pSTAT3/STAT3 | +3 (129/125) | +43 (156/109) | +86 (229/123) | +80 (237/132) |
| pAkt/Akt | +19 (130/109) | +3 (109/106) | +7 (118/110) | -13 (110/127) |
| pERK 1/2/ERK1/2 | +3 (105/102) | +3 (121/118) | -23 (91/119) | -69 (35/121) |

MDA

| Protein/activation vs. control (%) | gAd | 15’ | 30’ | 60’ |
| --- | --- | --- | --- | --- |
| pAMPK/AMPK | - 37 (79/126) | -24 (106/139) | -25 (78/120) | -29 (87/122) |
| pSTAT3/STAT3 | + 82 (215/118) | +81 (245/135) | +102 (233/115) | -11 (109/122) |
| pAkt/Akt | + 29 (101/78) | + 10 (106/96) | + 59 (113/71) | + 42 (135/95) |
| pERK 1/2/ERK1/2 | - 12 (89/91) | - 51 (65/133) | - 93 (6/85) | - 89 (11/93) |

LN18

| Protein/activation vs. control (%) | gAd | 15’ | 30’ | 60’ |
| --- | --- | --- | --- | --- |
| pAMPK/AMPK | + 33 (125/94) | - 3 (106/109) | - 29 (105/147) | - 5 (113/118) |
| pSTAT3/STAT3 | - 69 (30/96) | - 29 (62/87) | - 79 (19/90) | - 73 (33/120) |
| pAkt/Akt | - 8 (58/63) | + 24 (26/21) | + 79 (34/19) | - 12 (30/34) |
| pERK 1/2/ERK1/2 | - 22 (78/99) | - 11 (67/75) | - 13 (73/84) | 0 (100/100) |

The cells were treated with either gAd or ADP 355, as described in Figure 4 legend. The activation of proteins % increase vs. control and was measured by densitometry scanning of WB (phospho levels/total levels), as described in Materials and Methods. The phosphorylation status (phospho levels/total levels) of proteins in control cells is taken as 100%. The results are average from at least 2 experiments.
